# Plasma ion implantation enabled bio-functionalization of PEEK improves osteoblastic activity

**DOI:** 10.1063/1.5010346

**Published:** 2018-06-05

**Authors:** Edgar A. Wakelin, Giselle C. Yeo, David R. McKenzie, Marcela M. M. Bilek, Anthony S. Weiss

**Affiliations:** 1 Applied and Plasma Physics, School of Physics, The University of Sydney, NSW 2006, Australia; 2 School of Life and Environmental Sciences, The University of Sydney, NSW 2006, Australia; 3 Charles Perkins Centre, The University of Sydney, NSW 2006, Australia; 4 School of Aerospace, Mechanical and Mechatronic Engineering, The University of Sydney, NSW 2006, Australia; 5 The University of Sydney Nano Institute, The University of Sydney, NSW 2006, Australia; 6 Bosch Institute, The University of Sydney, NSW 2006, Australia

## Abstract

Slow appositional growth of bone *in vivo* is a major problem associated with polyether ether ketone (PEEK) based orthopaedic implants. Early stage promotion of osteoblast activity, particularly bone nodule formation, would help to improve contact between PEEK implantable materials and the surrounding bone tissue. To improve interactions with bone cells, we explored here the use of plasma immersion ion implantation (PIII) treatment of PEEK to covalently immobilize biomolecules to the surface. In this study, a single step process was used to covalently immobilize tropoelastin on the surface of PIII modified PEEK through reactions with radicals generated by the treatment. Improved bioactivity was observed using the human osteoblast-like cell line, SAOS-2. Cells on surfaces that were PIII-treated or tropoelastin-coated exhibited improved attachment, spreading, proliferation, and bone nodule formation compared to cells on untreated samples. Surfaces that were both PIII-treated and tropoelastin-coated triggered the most favorable osteoblast-like responses. Surface treatment or tropoelastin coating did not alter alkaline phosphatase gene expression and activity of bound cells but did influence the expression of other bone markers including osteocalcin, osteonectin, and collagen I. We conclude that the surface modification of PEEK improves osteoblast interactions, particularly with respect to bone apposition, and enhances the orthopedic utility of PEEK.

## INTRODUCTION

High performance organic polymers are an emerging alternative to titanium based orthopedic implants.^1^ Traditional metallic orthopedic devices risk early implant failure due to their high stiffness, resulting in bone degradation via stress shielding arising from a modulus discontinuity between the implant and the surrounding bone.^2,3^ Attempts to improve the cell-surface interactions of titanium and zirconium surfaces have been investigated through modifications of the physical surface structure with some success.^4,5^ These materials, however, continue to suffer from inappropriate bulk material properties. Polymeric implants provide the prospect of an isoelastic implant-tissue interface, significantly reducing the risk of stress shielding.^6^ Polyether ether ketone (PEEK) is a promising candidate for the next generation of orthopedic implant materials because of its bone-like mechanical properties^7–11^ and outstanding thermal^12^ and chemical stabilities.^13–16^ However, while well-tolerated *in vivo*, PEEK is mildly hydrophobic and bio-inert, resulting in poor surface tissue bonding, particularly in a bone apposition setting.^14,15,17^ This inadequate integration between the polymer implant surface and bone tissue often leads to implant failure. There is a clear need for surface modification technology that can improve the ability of PEEK to promote bone cell adhesion, growth, and associated bone formation.

Modification and functionalization of PEEK for bio-interfacing applications have been achieved using a number of techniques.^18–21^ Physical methods such as extruding PEEK with surface topology that mimics the physical geometry of trabecular bone, or with physiologically relevant pores, have enhanced cell attachment, proliferation, and mineralization.^19,22^ However, such methods do not improve the inherent biochemical properties of the PEEK surface. While plasma surface modification has been shown to improve the hydrophilicity^23^ and biological properties of PEEK,^20^ this modification lacks stability due to the re-orientation of the generated polar groups into the bulk material.^24^ Plasma treatment combined with metallic coatings such as gold represents a more permanent modification;^25^ however, such treatments require multi-step processes that are difficult to scale and costly.

Functionalizing cell-interfacing surfaces with a cell-modulatory protein can mask the underlying inert substrate and improve the biological properties of polymers.^26^ The conformation and biological activity of the protein, however, are dependent on the chemical and physical environment of the surface. In particular, a hydrophobic surface may change the conformation of the bound protein to an inactive state.^27^ Furthermore, simple physisorption results in a relatively weak attachment to the surface and therefore renders the bound protein prone to displacement by competing proteins in the surrounding aqueous medium or corrosive environments.^28,29^ In contrast, covalent immobilization prevents protein exchange, offering improved stability and persistence compared to physisorption.

Wet chemical techniques used to immobilize proteins to surfaces, such as silanization or carboxylic modification, require multiple step chemical reactions that create waste disposal issues, may leave toxic residuals, and are difficult to replicate at the industrial scale.^30,31^ Plasma immersion ion implantation (PIII) of organic polymers is an emerging dry industry-scalable technique for single-step protein immobilization without the need for linker chemistry.^32^ During PIII treatment, ions from plasma are accelerated toward the polymer by application of a pulsed bias to a surrounding conducting mesh. The ions bombard the polymer surface, breaking bonds and disrupting polymer chains. As the bonds reform, reactive unpaired electrons are generated within the modified region.^33,34^ These unpaired electrons are present in radicals that diffuse throughout the polymer to covalently react with molecules at the surface,^35^ such as atmospheric oxygen^36^ or biomolecules in solution.^37^

PEEK, PIII treated under identical conditions as utilized in this study, exhibits dynamic properties characterized by progressive oxidation of the surface^36^ and a decreasing concentration of radicals.^35^ These dynamic surface properties stabilize after 7 days to produce a stable hydrophilic surface.^36^ Due to its increased surface hydrophilicity, modified chemical structure, and radical content, PIII treated PEEK has been associated with improved cell interactions.^38,39^ Previous studies of PIII treated PEEK with nitrogen plasma have shown improved hydrophilicity and anti-bacterial and cell interaction properties in general^21,40,41^ but have not investigated functionalization of the surface for specific applications. We seek to build on the previous material characterization of PIII treated PEEK and expand its biological capabilities by immobilizing an active biomolecule to specifically induce bone nodule growth and development. This type of surface modification can easily be applied to PEEK materials with already optimised surface topology as it does not require surface flatness. We utilize tropoelastin, an extracellular matrix protein, which possesses strong cell signaling properties and has been shown to promote the attachment, spreading, proliferation, and activity of a number of cell types.^42^ Tropoelastin has also previously been shown to improve bone cell interactions on titanium and zirconium surfaces.^43^ In this study, we investigate the potential of tropoelastin-functionalized PIII treated PEEK for orthopedic applications. We demonstrate greatly improved performance of human osteoblast-like osteosarcoma cells (SAOS-2), a model osteoblast cell line,^44^ on the modified PEEK surfaces, as characterized by significantly increased cell adhesion, growth, and activity, leading to enhanced bone matrix maturation and mineralization.

## RESULTS AND DISCUSSION

### Tropoelastin saturation and covalent immobilization on PIII treated PEEK

To confirm that the PIII treated surfaces were suitable for active protein immobilization, surfaces PIII treated for 60 to 1600 s were incubated in 0 to 50 *μ*g/ml tropoelastin. Fourier transform infrared (FTIR) and enzyme-linked immunosorbent assay (ELISA) measurements^49,50^ were used to confirm that a monolayer of tropoelastin was covalently attached to the PEEK surfaces following PIII treatment (details are given in the supplementary material and Fig. S1 in the supplementary material). While ELISA will only detect a surface layer due to the steric hindrance of antibody molecules, FTIR can probe surface thicknesses on the order of 1 *μ*m. As these methods saturate at the same tropoelastin concentration, it can be inferred that multilayered tropoelastin structures do not form on the surface and that only a protein monolayer is present. Covalent tropoelastin attachment saturated at a coating concentration of 5 *μ*g/ml and a PIII treatment time of 120 s. Tropoelastin immobilization to PIII treated surfaces is achieved through reactions with radicals generated by the PIII treatment on the surface.^37^ The generation and final concentration of radicals present in a sub-surface layer are functions of ion fluence, which in turn are functions of treatment time.^36^ To ensure that the surfaces were sufficiently activated with radicals and coated with a full monolayer of tropoelastin, a PIII treatment time of 800 s and a tropoelastin coating concentration of 20 *μ*g/ml were selected for subsequent experiments.

### SAOS-2 attachment and spreading

**FIG. 1.**
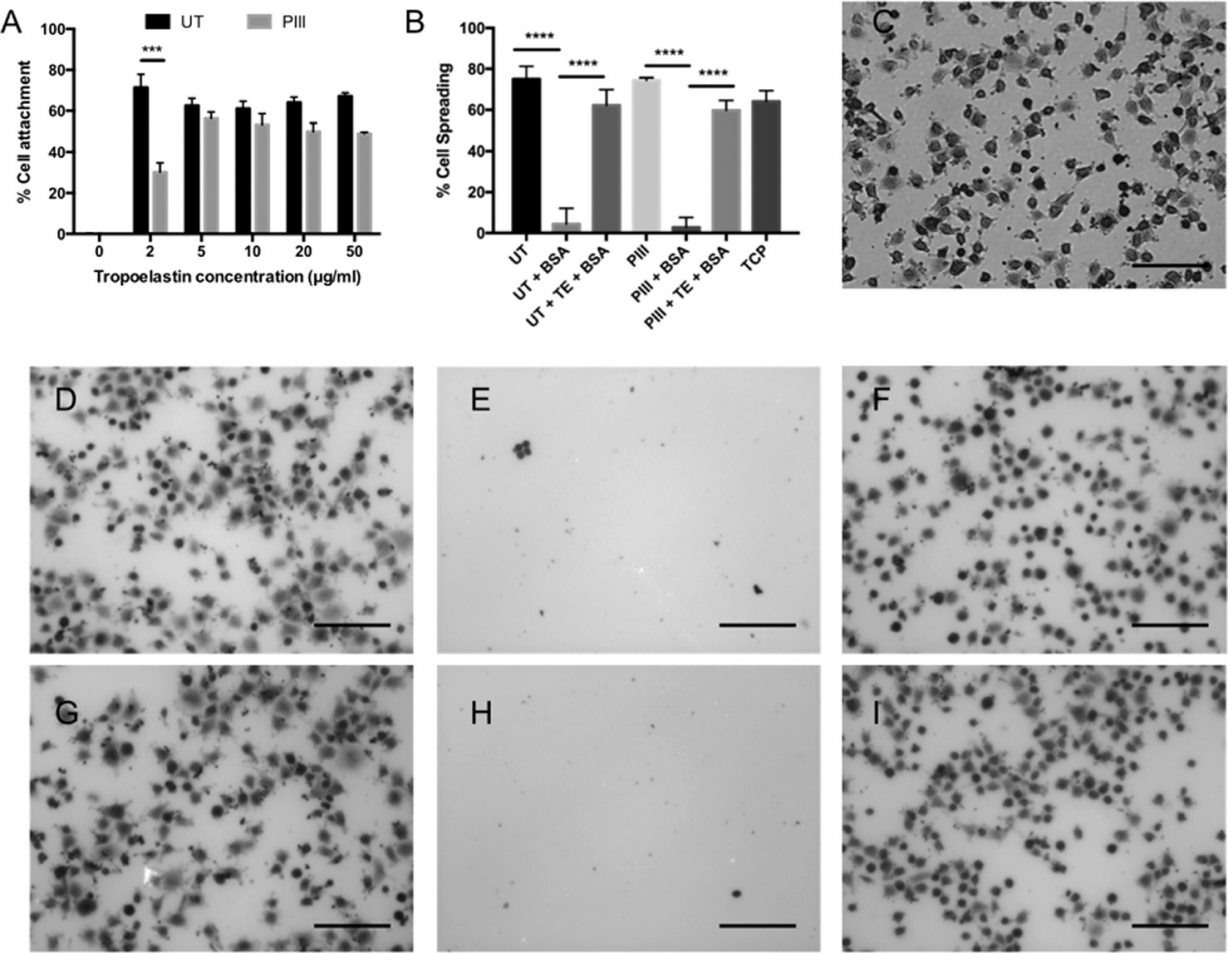
(a) SAOS-2 cell attachment on untreated and PIII treated PEEK coated with increasing concentrations of tropoelastin and blocked with denatured BSA. (b) SAOS-2 cell spreading on bare, BSA blocked, or tropoelastin coated (incubated in 10 *μ*g/ml tropoelastin solution) and BSA blocked untreated and PIII treated PEEK surfaces, as well as tissue culture plastic (TCP) controls. (c)–(i) Representative images of spread and unspread cells on (c) TCP, (d) bare untreated PEEK, (e) untreated PEEK+BSA, (f) untreated PEEK+tropoelastin (TE)+BSA, (g) bare PIII treated PEEK, (h) PIII treated PEEK+BSA, and (i) PIII treated PEEK+TE+BSA. The scale bar represents 100 *μ*m.

FTIR and ELISA measurements show that tropoelastin adsorbs on and covalently attaches to PIII treated PEEK surfaces. Biomolecule coated surfaces, unlike surfaces with modified topology, have the potential to stimulate specific and targeted cell responses on the surface. However, to confer bio-functionality to these materials, surface-bound tropoelastin must retain its cell binding and signaling activity. The extent of SAOS-2 attachment on untreated and PIII treated PEEK coated with 0 to 50 *μ*g/ml tropoelastin and bovine serum albumin (BSA) blocked is shown in Fig. 1(a). No cell attachment was observed on surfaces without tropoelastin, indicating complete BSA coverage of the underlying PEEK material such that any cellular interaction occurs directly and solely with the tropoelastin layer. Cell adhesion on tropoelastin-coated untreated and PIII treated PEEK surfaces confirmed that the protein was functional when immobilized on both surfaces. PIII treated surfaces incubated with increasing amounts of tropoelastin supported a cell attachment profile that closely reflected the extent of tropoelastin binding and reached a maximum cell attachment of 56 ± 5% on surfaces with full tropoelastin coverage. Significantly higher cell binding was observed on untreated PEEK (71 ± 11%) compared to PIII treated PEEK (30 ± 8%) at low coating concentrations (2 *μ*g/ml), indicating that a higher proportion of surface-bound molecules are presented in a functional conformation when adsorbed on the untreated surface. The positively charged C-terminus of tropoelastin has previously been identified as a powerful cell binding region,^51^ which, if obscured, may reduce its cell binding activity at low concentrations. PIII treated organic polymers possess a negative surface charge,^52^ generating an environment where the C-terminus will be attracted to the PIII treated PEEK surface and therefore unable to interact with cells. Tropoelastin adsorbed on untreated PEEK, however, will have a reduced orientation preference on the surface, thereby displaying a greater number of cell binding motifs. This effect saturates at 5 *μ*g/ml tropoelastin, after which the cell binding activities are equal. Physisorbed tropoelastin molecules, however, represent an inherently unstable surface in which tropoelastin is not covalently immobilized and may be displaced by other proteins *in vivo* through processes such as the Vroman effect.^29^

SAOS-2 cell spreading on untreated and PIII treated PEEK is shown quantitatively in Fig. 1(b). Bare untreated and PIII PEEK surfaces show high levels of cell spreading (75 ± 6% and 74 ± 3%, respectively) similar to those observed on tissue culture polystyrene (TCP) (64 ± 5%). These surfaces, however, can indiscriminately bind other proteins *in vivo*, which may not possess cell adhesive or spreading capabilities. For example, surfaces coated with denatured BSA exhibited minimal levels of spreading. Surfaces coated with tropoelastin, however, supported high levels of cell spreading; 62 ± 8% and 60 ± 6% of cells were spread on protein-coated untreated and PIII treated PEEK, respectively. Compared to the bare surfaces which allow non-specific protein adhesion *in vivo*, PIII treated tropoelastin coated surfaces represent a robust, stable environment for cell spreading that is unlikely to be functionally compromised by contact with non-cell-adhesive molecules. Representative images of cell spreading on TCP and PEEK samples are shown in Figs. 1(c)–1(i). Few cells adhered to the BSA coated surfaces, and they appeared rounded, indicating lack of spreading. In contrast, there was a high abundance of cells on the tropoelastin-coated surfaces, the majority of which displayed protruding lamellipodia characteristic of spread cells.

### SAOS-2 proliferation

**FIG. 2.**
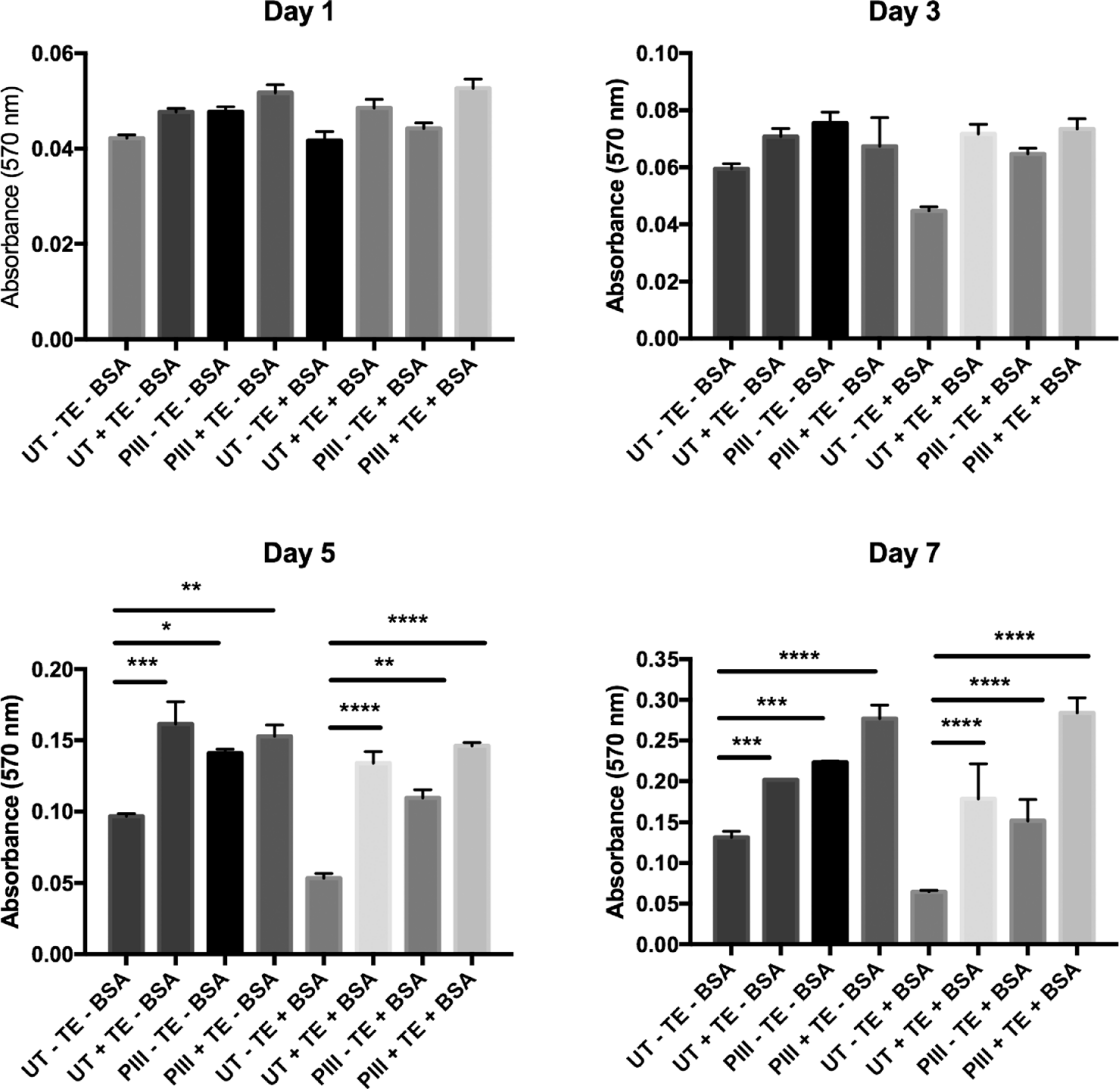
Proliferation of SAOS-2 cells over 7 days on bare and tropoelastin (TE) coated untreated and PIII treated PEEK samples with and without BSA blocking.

SAOS-2 cell numbers increased on bare and tropoelastin coated untreated and PIII treated PEEK surfaces, with and without BSA blocking, over 7 days, as shown in Fig. 2. Significant differences in the extent of cell proliferation were observed at 5 and 7 days post-seeding. Bare PIII treated PEEK promoted 134 ± 39% and 70 ± 8% higher proliferation at day 7 compared to the untreated surface with and without BSA blocking, respectively. This significant increase indicates that the improved hydrophilicity and chemical modification previously reported as a result of PIII treatment of PEEK^36^ create an improved cytocompatible environment. By day 7, the tropoelastin coated surfaces further augmented cell proliferation by 54 ± 5% over the unblocked untreated PEEK, 176 ± 82% over BSA-blocked untreated PEEK, 24 ± 2% over unblocked PIII treated PEEK, and 83 ± 27% over BSA-blocked PIII treated PEEK. Among all samples, the PIII treated tropoelastin coated samples induced the highest proliferation response. The nearest competitor for the tropoelastin coated surface is the unblocked PIII treated surface (46 ± 10% and 70 ± 19% enhanced proliferation over the untreated unblocked surface at days 5 and 7, respectively); however, this performance may be modulated *in-vivo* by the adsorption and immobilization of serum proteins prior to cell seeding. We attempted to simulate this scenario by blocking with heat denatured BSA prior to cell seeding, and note that the PIII treated surface still provides greatly enhanced cell proliferation over the untreated surface (105 ± 41% at day 5 and 134 ± 68% at day 7) when these surfaces are exposed to BSA prior to cell seeding.

Radical quenching during ageing reduces the ability of the surface to immobilize serum proteins, such that after long ageing times, the PIII treated surface will more closely resemble a hydrophilic untreated surface that does not irreversibly immobilize serum proteins and therefore allows protein exchange.^35^ Immobilizing biomolecules after short ageing times, however, ensures homogenous and robust surface coverage. Therefore, tropoelastin coating of the PIII treated surface is advantageous not only in promoting a greater degree of osteoblast-like cell proliferation but also in maintaining the functional stability of the material.

### SAOS-2 ALP activity

**FIG. 3.**
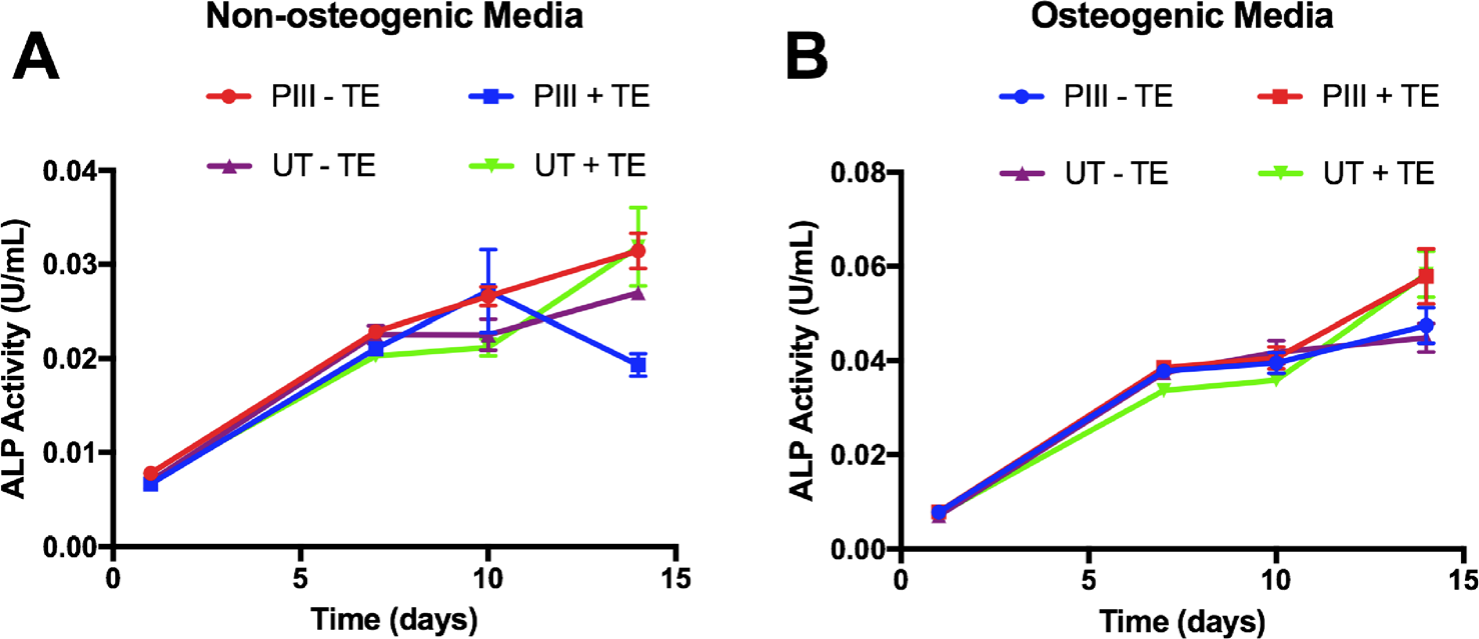
Alkaline phosphatase (ALP) activity by SAOS-2 cells on bare and tropoelastin coated untreated and PIII treated PEEK cultured for 14 days in (a) media without osteogenic supplements and (b) media with osteogenic supplements.

Extracellular alkaline phosphatase (ALP) production by SAOS-2 cells was unaffected by both PIII surface treatment and tropoelastin coating over 15 days (Fig. 3). ALP production, however, significantly increased in all cases when samples were cultured in osteogenic media after 7, 10, and 15 days post confluence. We therefore concluded that the surface treatment and tropoelastin coating did not interfere with ALP production in either environment, allowing for the natural osteogenic progression of bound cells.

SAOS-2 cells express high levels of ALP in proportion to cell density and are insensitive to most external stimuli except specific osteogenic supplements.^44^ For example, SAOS-2 ALP activity has been found to be unresponsive to 1,25-dihydroxyvitamin D_3_, a steroid hormone typically capable of stimulating ALP activity in other human osteosarcoma cell lines (e.g., SAOS-1), suggesting that the enzyme is constitutively expressed at near maximum levels.^53^ Improved surface chemistry and biological activity are therefore unlikely to increase SAOS-2 ALP expression, regardless of the substrate molecular environment, as observed here.

### Bone marker expression

**FIG. 4.**
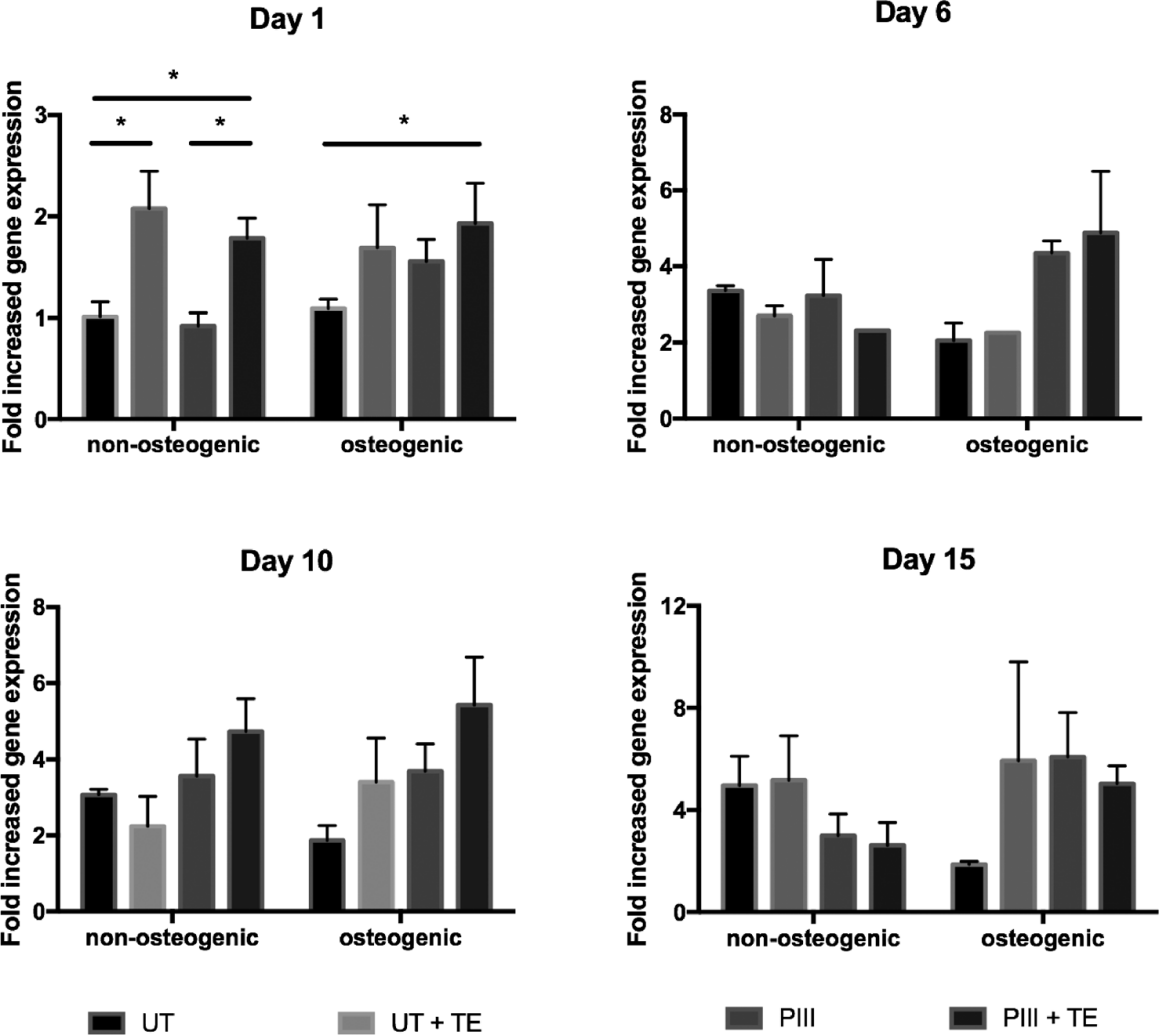
ALP gene expression of SAOS-2 cells cultured on bare or tropoelastin coated untreated and PIII treated PEEK in normal or osteogenic media, as measured by qPCR over 15 days post-confluence.

SAOS-2 cells cultured post-confluence on the PEEK surfaces expressed common bone markers including ALP, Collagen 1 (COL1), osteocalcin (OCN), and osteopontin (OPN). ALP gene expression of cells cultured on tropoelastin coated untreated and PIII treated PEEK, shown in Fig. 4, was significantly increased at day 1 post-confluence. At this early time point, cells on surfaces coated with tropoelastin and cultured in non-osteogenic media exhibited a 2-fold increase in ALP expression compared to uncoated surfaces. A similar trend was observed with samples cultured in osteogenic media. This tropoelastin-mediated increase in ALP upregulation was short-term, as transcript levels were comparable among samples after day 1, consistent with their similar levels of ALP protein secretion.

**FIG. 5.**
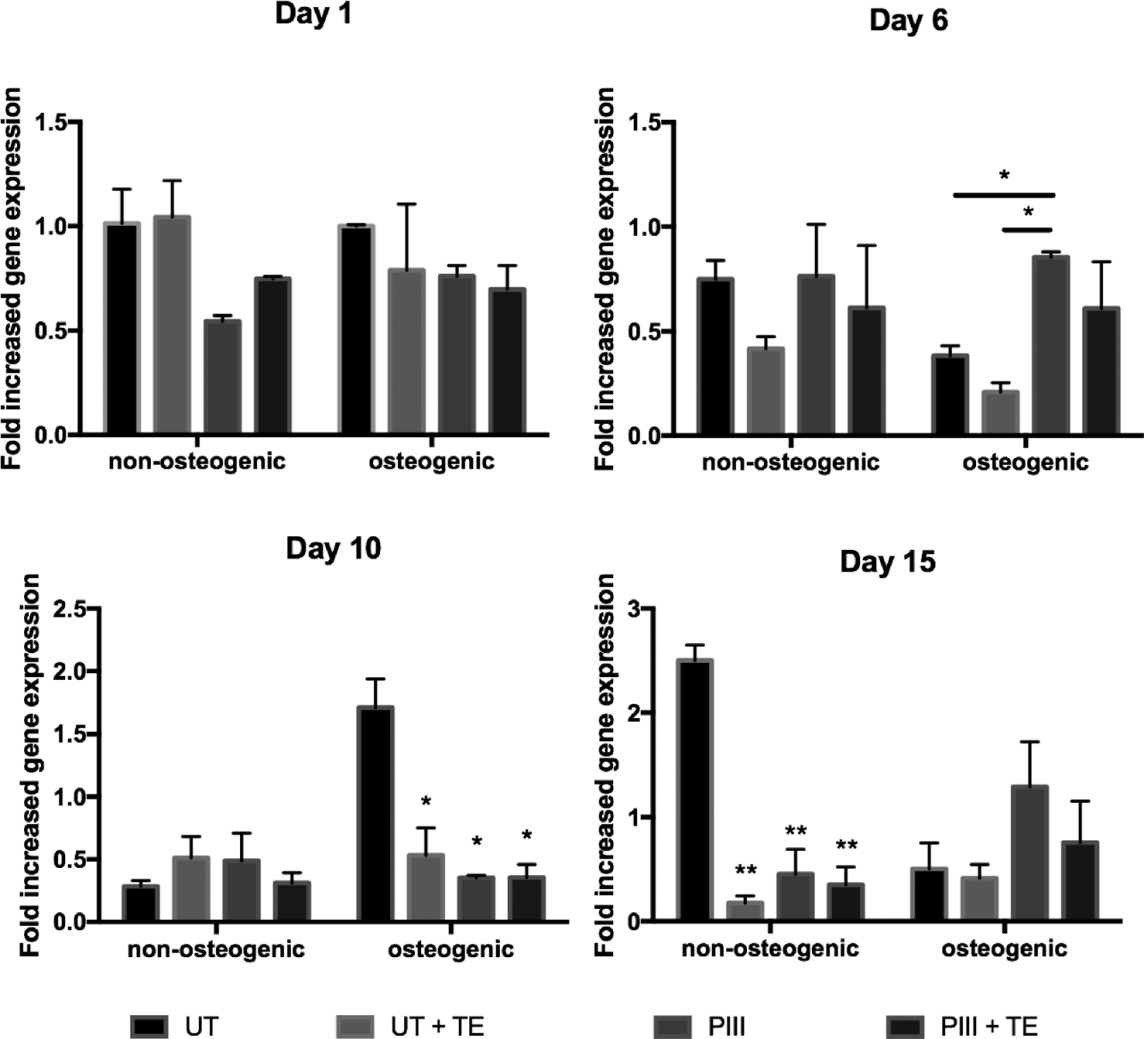
Collagen 1 (COL1) gene expression of SAOS-2 cells as measured by q-PCR.

Figure 5 shows the change in COL1 expression of SAOS-2 cells cultured on untreated and PIII treated PEEK measured over 15 days. COL1 expression by cells on PIII treated PEEK samples remained relatively constant over the culture time period. Cells cultured on untreated PEEK without tropoelastin and in osteogenic media exhibited a 60 ± 9% reduction in COL1 expression at day 6 and 1.6-fold upregulation at day 10. The same sample cultured in non-osteogenic media also exhibited a later 2.5-fold upregulation of COL1.

COL1 expression in osteoblast cells reaches a maximum during proliferation, followed by gradual downregulation.^54^ SAOS-2 cells, however, show high levels of COL1 gene expression which are independent of confluency.^55^ The unexpected COL1 upregulation at late time points may be due to the comparative hydrophobicity of the untreated PEEK surface compared to PIII treated PEEK,^36^ stimulating increased extracellular matrix formation for surface coverage. This COL-1 upregulation is not observed on the PIII treated or tropoelastin-coated surfaces, presumably because they constitute a more biologically compatible hydrophilic environment.

**FIG. 6.**
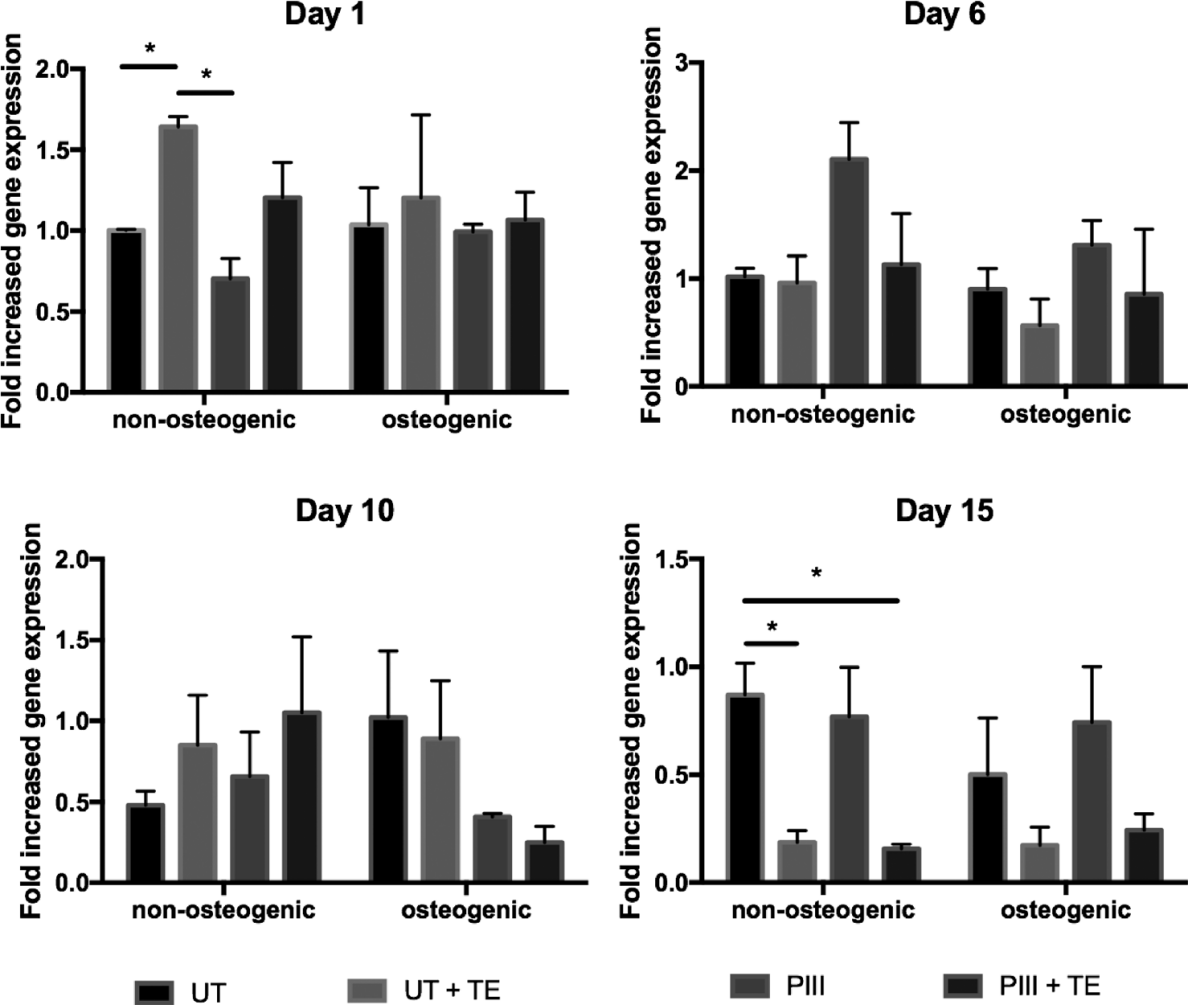
Osteocalcin (OCN) gene expression of SAOS-2 cells as measured by q-PCR.

Figure 6 shows the change in OCN gene expression of SAOS-2 cells cultured on untreated and PIII treated PEEK. A reduction in OCN expression by 89 ± 6% was seen on tropoelastin coated surfaces cultured in non-osteogenic media by the end of the incubation period.

OCN is generally expressed late in bone development and is involved in mineral deposition.^56^ SAOS-2 cells, however, display a highly mature osteoblastic profile, including early expression of OCN.^55^ Early upregulation was therefore expected and observed at day 1 to be triggered by the presence of tropoelastin. SAOS-2 cells have been shown to lack cell density dependent gene expression,^55^ suggesting that the OCN downregulation at late time points is unlikely to be associated with overconfluence. OCN downregulation was also unlikely due to the PIII treatment, as it only occurred on the tropoelastin coated surfaces. OCN downregulation was therefore most likely due to tropoelastin on the surface through an unknown mechanism.

**FIG. 7.**
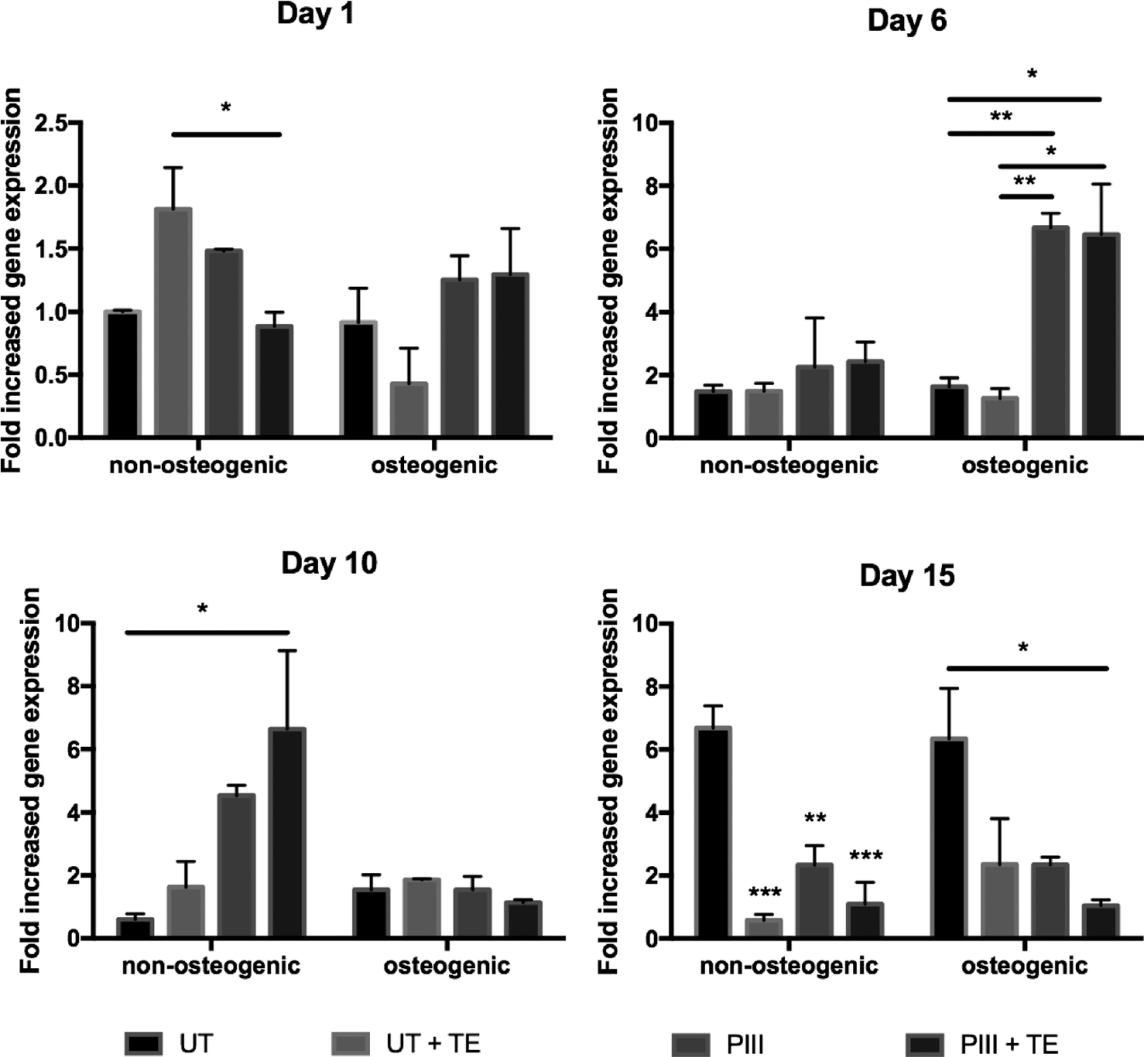
Osteopontin (OPN) gene expression of SAOS-2 cells as measured by qPCR.

Figure 7 shows the change in OPN expression of SAOS-2 cells cultured on untreated and PIII treated PEEK. OPN expression exhibited short-term stimulation at variable time points on the different surfaces before a return to basal levels. Cells on PIII treated PEEK, both with and without tropoelastin coating, exhibited upregulation (6-fold increase) at day 6 post-confluence in osteogenic media, and at day 10 post-confluence in non-osteogenic media, up to 9 days earlier than untreated surfaces. Cells on bare, untreated PEEK also showed a 6-fold increase in expression but only by day 15 post-confluence in both osteogenic and non-osteogenic media.

Osteoblast OPN gene expression is up-regulated in the presence of dexamethasone and high calcium concentrations and is linked to increased bone nodule deposition.^57^ This is consistent with our observation of accelerated upregulation of OPN in cells cultured in osteogenic media containing dexamethasone. Upregulation of OPN in cells cultured under non-osteogenic conditions was likely triggered by a high local calcium concentration at the bone nodules. This effect occurred at later time points due to the slower bone nodule formation in non-osteogenic conditions.

### Bone nodule formation

**FIG. 8.**
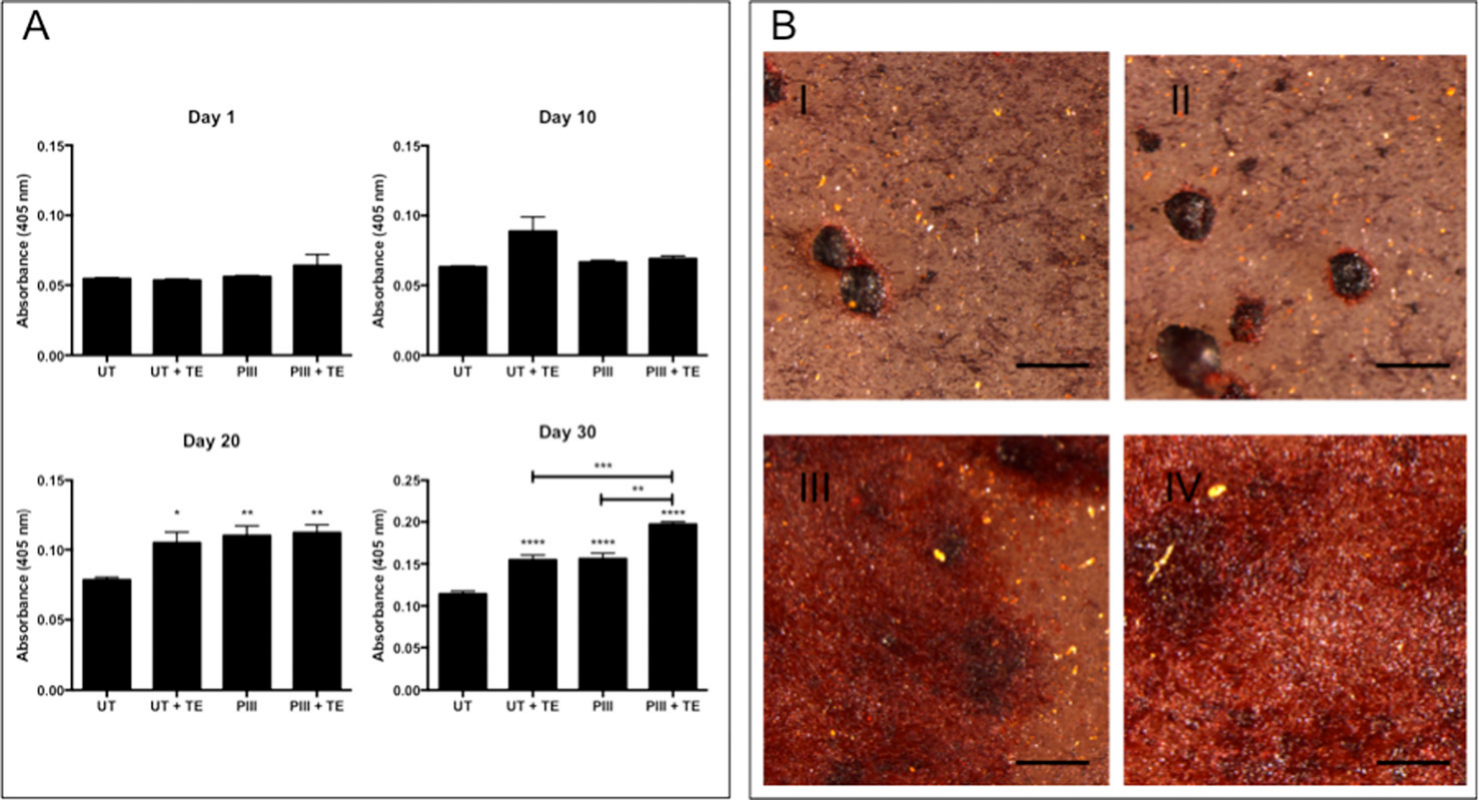
(a) Calcified bone nodule formation by SAOS-2 cells as measured by staining with Alizarin red on bare and tropoelastin-coated untreated and PIII treated surfaces cultured over 30 days in media with osteogenic supplements. Asterisks refer to a significant difference compared to the untreated PEEK without osteogenic supplements. (b) Typical morphology of bone nodule growth by SAOS-2 cells cultured in osteogenic media on (I) untreated PEEK, (II) untreated PEEK incubated in tropoelastin, (III) PIII treated PEEK, and (IV) PIII treated PEEK incubated in tropoelastin. The scale bar indicates 500 *μ*m.

SAOS-2 bone nodule formation was measured over 30 days by staining calcium deposits with Alizarin red (Fig. 8). Cells grown on PIII treated samples displayed higher levels of bone nodule production than those on untreated samples after 20 days in culture with osteogenic supplements. The benefit of tropoelastin coating was evident at later time points, as demonstrated by the more extensive bone nodule formation on tropoelastin-coated surfaces after 30 days in culture. Cells on PIII treated, tropoelastin coated PEEK exhibited 58 ± 6% increased calcium deposition over cells on untreated, uncoated PEEK.

Representative images of the stained nodules in Fig. 8(b) showed markedly different morphologies between the untreated and PIII treated PEEK samples 30 days post-confluence. Cells on untreated PEEK surfaces formed discrete calcified nodules separated by large voids. Cells on tropoelastin-coated untreated PEEK displayed nodules with the same morphology but at a higher abundance. Cells on PIII treated surfaces, however, exhibited more homogenous calcification with a greater nodule-surface contact area. Bone nodule growth *in vitro* can have a widely varying morphology,^58^ where high nodule formation resulting in a homogenous layer is typical of a more hydrophilic bioactive surface.^59,60^ Cells on tropoelastin-coated PIII treated PEEK produced similarly homogeneous bone nodules and at a higher density.

We propose a model where the oxidation of the PEEK surface after PIII treatment and the associated increase in the polar surface energy^36^ creates a more hydrophilic bio-interface, producing an environment more favorable for bone nodule formation.^59,60^ Tropoelastin significantly improves cell binding and proliferation,^42^ which ultimately translates to increased bone mineralization. The low elastic modulus of tropoelastin does not adversely affect nodule growth as the monolayer coating applied here is only several nanometers thick but instead promotes cell proliferation and early upregulation of osteoblastic markers ALP and OPN. Combining these effects with the benefits of PIII treatment leads to a further enhanced osteogenic environment superior to that of bare PEEK even in osteogenic media.

## CONCLUSIONS

Plasma immersion ion implantation (PIII) and subsequent covalent immobilization of tropoelastin is a simple and scalable two-step bio-functionalization process shown here to significantly improve the bio-activity of PEEK. This functionally stable, biologically active surface significantly improved osteoblast-like cell attachment, spreading, proliferation, osteoblastic activity, and bone nodule formation compared to unmodified bare PEEK and surfaces with either PIII treatment or protein coating alone. Improving bone apposition on PEEK surfaces significantly enhances the utility of isoelastic polymer based-orthopedic implantable devices, addressing a major risk factor in current prostheses. Furthermore, this functionalization process may be extended to other organic polymers and bio-molecules to generate bio-active environments tailored to induce specific cell responses.

## METHODS

### Materials

Medical grade semi-crystalline PEEK with a sheet thickness of 220 *μ*m and a density of 1301 kg m^−3^ was obtained from Victrex Manufacturing Ltd., Lancashire, UK. PEEK sheets were cut into 0.6 × 0.8 cm or 1.3 × 1.0 cm samples for biological testing. Recombinant human tropoelastin was obtained from Elastagen Pty Ltd, Australia. All reagents were obtained from Sigma Aldrich unless otherwise indicated.

### PIII treatment

Samples were PIII treated for 60–1600 s, corresponding to ion fluences of 7.5 × 10^14^–2 × 10^16^ ions cm^−2^. Treatment times were chosen to represent three ranges of fluence: low fluence where characteristics of the treated surface vary greatly with ion fluence (60–240 s or 7.5 × 10^14^–3 × 10^15^); medium fluence where characteristics of the treated surface evolve slowly with ion fluence (400–800 s or 5 × 10^15^–10^16^); and high fluence where characteristics of the treated surface no longer vary with ion fluence (1600 s or 2 × 10^16^).^11^ The nitrogen plasma consisting mainly of N_2_^+^ and N^+^ ions, and neutral gas species was generated with an rf power of 100 W at a pressure of 2 × 10^−3 ^Torr and guided towards the sample by use of magnetic field coils. Ions were accelerated with a pulsed bias voltage of −20 kV for a pulse length of 20 *μ*s applied at 50 Hz. Samples for all cell studies were PIII treated for 800 s.

### Tropoelastin coating

PIII treated samples were aged for 1 week before protein coating. Untreated and PIII treated PEEK were incubated in 2 to 50 *μ*g/ml tropoelastin solution overnight at 4 °C. Samples for cell assays were coated with 20 *μ*g/ml tropoelastin. Samples were then washed three times with phosphate buffered saline (PBS) to remove the unbound protein.

### Enzyme-linked immunosorbent assay (ELISA)

Tropoelastin-coated samples were blocked with 3% (w/v) bovine serum albumin (BSA) for 1 h at room temperature. A surface-bound protein was detected with a 1:2000 BA-4 mouse anti-elastin primary antibody, followed by a 1:5000 goat anti-mouse-horseradish-peroxidase-conjugated secondary antibody. Samples were then incubated in 2,2′-azino-bis(3-ethylbenzothiazoline-6-sulphonic acid) (ABTS) substrate solution (1.04 mg/ml 2,2′-azino-bis-3-ethylbenzothiazoline-6-sulfonic acid diammonium salt, 0.05% (v/v) H_2_O_2_, 10 mM CH_3_COONa, and 5 mM Na_2_HPO_4_) for 45 min at room temperature, after which the absorbance was measured at 405 nm.

### Fourier transform infrared (FTIR) spectroscopy and SDS washing

FTIR spectra of PEEK samples incubated in tropoelastin were obtained using a Digilab FTS 7000 spectrometer fitted with an attenuated-total-reflection trapezium germanium crystal at an angle of incidence of 45°. Spectra were averaged over 500 scans with a resolution of 4 cm^−1^. A background scan was obtained between each sample scan, and the spectra were normalized against an uncoated PEEK sample after the subtraction of the background water vapor spectrum. The presence of the protein was detected by comparison of the amide A (3250 cm^−1^), amide I (1650 cm^−1^), and amide II (1545 cm^−1^) peaks with a sample of PEEK not coated with tropoelastin.

Selected samples were washed with 5% (w/v) sodium dodecyl sulfate (SDS) in PBS at 80 °C for 10 min to remove the non-covalently bound protein,^32,37,45,46^ then rinsed in Milli-Q water, and dried before obtaining a post-SDS FTIR spectrum.

### Cell culture

SAOS-2 cells were obtained from Sigma Aldrich and cultured in McCoy's 5A media with 10% (v/v) fetal bovine serum (FBS) (Life Technologies) and 1% (v/v) l-glutamine (Lonza). Cells were harvested from culture flasks with trypsin-EDTA. Selected samples were cultured in media with osteogenic supplements (1 mM sodium glycerophosphate, 0.17 mM l-ascorbic acid, and 10 nM dexamethasone) where indicated. Ethics approval is not required for this study.

### Sterilization

Samples for cell assays were exposed to ultraviolet light for 1 h prior to protein coating and cell seeding.

### Cell attachment

Bare and functionalized PEEK samples were blocked with 1% (w/v) heat denatured BSA in PBS for 1 h at room temperature. BSA was heat denatured at 80 °C for 10 min and then cooled on ice. Blocked samples were then washed 3 times with PBS.

SAOS-2 cells were seeded in a serum-free medium on the PEEK surfaces at a concentration of 2.5 × 10^5^ cells/cm^2^. To quantify the percentage of cell attachment to the samples, a standard curve was generated from cells seeded on PIII treated, unblocked PEEK at 0%, 50%, and 100% of the seeding density used above. All samples were then incubated at 37 °C in 5% CO_2_ for 1 h to allow for cell attachment. Samples (excluding the standards) were then washed 2 times with PBS to remove non-adherent cells. Samples and standards were then fixed with 3% (w/v) formaldehyde for 20 min at room temperature. All samples were washed 3 times with PBS and stained with 0.1% (w/v) crystal violet in 0.2 M 2-(N-morpholino)ethanesulfonic acid buffer, pH 5.0 at room temperature for 1 h. Samples were then washed 3 times with Milli-Q water to remove excess stain. The stain was solubilized with 10% (v/v) acetic acid, and the absorbance was measured at 570 nm to quantify cell attachment.

### Cell spreading

Samples were prepared following the same protocol as the cell attachment assay. After cell fixation, samples were washed 3 times with MilliQ water and imaged with a Leica MZ16 FA stereoscope to determine the number of spread and unspread cells on the surface.

### Cell proliferation

Where indicated, samples were blocked with 1% (w/v) heat denatured BSA for 1 h at room temperature. The blocked samples were then washed 3 times with PBS.

SAOS-2 cells were seeded on the PEEK surfaces at a concentration of 1 × 10^4^ cells/cm^2^ and cultured at 37 °C in 5% CO_2_ for 7 days. On days 1, 3, 5, and 7 after seeding, samples were washed, fixed, stained, and quantified as described in the cell attachment protocol.

### Alkaline phosphatase (ALP) detection

SAOS-2 cells were seeded on PEEK samples, which were not BSA blocked, at a concentration of 5 × 10^4^ cells/cm^2^, and cultured in growth media at 37 °C in 5% CO_2_ until confluence. At confluence, the media in selected samples were augmented with osteogenic supplements for the duration of the assay. The media were changed every 48 h during culture and 24 h before testing for ALP activity.

At 1, 7, 10, and 14 days post-confluence, extracellular ALP activity was measured with an ALP assay kit (Abnova). The resulting absorbance was then quantified by comparison to a standard curve of known ALP activity.

### Real-time quantitative polymerase chain reaction (qPCR)

SAOS-2 cells were seeded on surfaces, which were not BSA blocked, at a concentration of 1 × 10^6^ cells/cm^2^, and incubated in growth media at 37 °C in 5% CO_2_ until confluence. At confluence, the media in selected samples were augmented with osteogenic supplements for the duration of the assay.

RNA was extracted from all samples at 1, 6, and 10 days post-confluence using a High Pure RNA Isolation kit (Roche). The RNA yield and purity were determined using a NanoDrop 2000c (Thermo Scientific). Complementary DNA (cDNA) synthesis was then performed using a Transcriptor First Strand cDNA Synthesis Kit (Roche). Samples were heated to 25 °C for 10 min, 55 °C for 30 min, and 85 °C for 5 min in a Mastercycler Nexus Gradient (Eppendorf). Real-time qPCR was then performed to investigate the change in transcript expression of collagen 1 (COL1), osteocalcin (OCN), osteopontin (OPN), and ALP, using glyceraldehyde-3-phosphate dehydrogenase (GAPDH) as a reference using a Lightcycler 480 II instrument (Roche). Forward and reverse primers were supplied by Sigma Aldrich (GAPDH, COL1, OCN, and ALP), using sequences described by Liskova *et al.*,^47^ shown in Table I, and Biorad (OPN). Transcript levels were presented as a fold increase over the day 1 post-confluence expression levels on the untreated bare PEEK surface.

**TABLE I.** Sequence of forward and reverse primers used in qPCR.

Primer	Forward primer (5′-3′)	Reverse primer (5′-3′)
GAPDH	TGCACCACCAACTGCTTAGC	GGCATGGACTGTGGTCATGAG
COL1	CAGCCGCTTCACCTACAGC	TTTTGTATTCAATCACTGTCTTGCC
OCN	GAAGCCCAGCGGTGCA	CACTACCTCGCTGCCCTCC
ALP	GACCCTTGACCCCACAAT	GCTGCTACTGCATGTCCCCT
OPN	Data not provided, Biorad assay ID: qHsaCID0012060	

### Bone nodule formation

Samples in osteogenic and non-osteogenic growth media were prepared as described for alkaline phosphatase detection assays. Bone nodule formation was measured by staining calcium rich deposits with Alizarin red^48^ at 1, 10, 20, and 30 days post-confluence, following the procedure described by Gregory *et al.*^48^

### Statistical analyses

The values were reported as the mean ± standard error (n = 3). The statistical significance was calculated using the analysis of variance, with a significance threshold of p ≤ 0.05. Asterisks in figures denote p-values as follows: *p ≤ 0.05, **p ≤ 0.01, ***p ≤ 0.005, and ****p ≤ 0.001.

## SUPPLEMENTARY MATERIAL

See supplementary material for the details of FTIR and ELISA measurements used to confirm the presence of a monolayer of tropoelastin covalently attached to PEEK surfaces following PIII treatment.
